# DNA replication and cell enlargement of *Enterococcus faecalis* protoplasts

**DOI:** 10.3934/microbiol.2019.4.347

**Published:** 2019-10-25

**Authors:** Satoshi Kami, Rintaro Tsuchikado, Hiromi Nishida

**Affiliations:** Department of Biotechnology, Toyama Prefectural University, 5180 Kurokawa, Imizu, Toyama 939-0398, Japan

**Keywords:** DNA replication, *Enterococcus faecalis*, novobiocin treatment, protoplast enlargement, replication initiation, replication termination

## Abstract

Protoplasts of *Enterococcus faecalis* did not divide but enlarged in Difco Marine Broth containing penicillin. Our previous studies have demonstrated that transcription and translation were essential for bacterial cell enlargement. However, it was uncertain whether replication was also essential. In this study, we measured the amount of DNA in *E. faecalis* cells during the course of enlargement using quantitative polymerase chain reaction. The growth of normally divided cells (native forms) of *E. faecalis* exhibited a log phase before 6 h of incubation was reached. Although a difference in quantitation cycle (Cq) values between the replication initiation and termination regions was observed in the log phase, it was not present in the stationary growth phase. On the other hand, the amount of DNA in *E. faecalis* protoplasts increased during the cell enlargement incubation. The difference of Cq values between the protoplasts at 0 and 96 h of incubation was 8–9, indicating that the DNA amount at 96 h was 200–500 times higher than that at 0 h. The Cq values differed between the replication initiation and termination regions, indicating that the replication level was high. When novobiocin, a DNA replication inhibitor, was added to the medium at 24 h of incubation, DNA replication and cell enlargement were almost stopped. Thus, replication plays an important role in the enlargement of *E. faecalis* protoplasts.

## 1. Introduction

In native forms of bacteria, precise chromosomal DNA replication occurs during cell growth and division. To transfer the genetic information (chromosomal DNA) by cell division, DNA replication is required, which is also associated with cell surface biosynthesis (cell size) [1]–[8].

Lysozyme-treated bacterial protoplasts do not undergo prolonged growth or division in the presence of an inhibitor of peptidoglycan biosynthesis [9]. To recover to their native forms, cell wall re-synthesis is necessary [10]. However, bacterial protoplasts and spheroplasts can enlarge in Difco Marine Broth (DMB) containing an inhibitor of peptidoglycan biosynthesis, for example, penicillin [11]–[15]. The calcium and magnesium ions in DMB as well as the processes of transcription and translation are essential for spheroplast enlargement [15]. DNA replication has been reported to occur during spheroplast enlargement [11],[12],[16],[17], and the RNA expression pattern of the enlarged spheroplasts differs from that of normally divided cells (native forms) [13]. For example, DNA replication of chromosome and plasmid occurred during the enlargement incubation of *Escherichia coli* spheroplasts [11]. Generally, DNA replication is essential before cell division. However, if a cell does not divide, this may be unnecessary for spheroplast enlargement. If a spheroplast possesses a sufficient amount of chromosomal DNA, transcription and translation could occur in the cell. Thus, we speculated that DNA replication was halted during spheroplast enlargement. When native forms are used, it is not determined whether the drug inhibits cell division or membrane synthesis. However, when protoplasts are used, the cells do not divide, so the influence on cell membrane synthesis can be estimated.

In this study, we used the Gram-positive, lactic acid bacterium *Enterococcus faecalis*. *Enterococcus* is an important bacterium for ecology, epidemiology, and virulence [18]. The genetic diversity *E. faecalis* has been demonstrated [19]. We measured the amounts of DNA present in protoplasts of *E. faecalis* NBRC 100480 (type strain of this species) at 0, 24, 48, 72, and 96 h of enlargement incubation using real-time quantitative (q) polymerase chain reactions (PCRs). Furthermore, we added novobiocin, an inhibitor of DNA replication, to the incubation medium at 24 h. Thereafter, we measured the amount of DNA in the cells and observed the degree of cell enlargement.

## 2. Methods

### 2.1. Preparation and culture of protoplasts

A single colony of *E. faecalis* NBRC 100480 was streaked on a de Man, Rogosa, and Sharpe (MRS) agar plate (10 g/L peptone, 10 g/L beef extract, 5.0 g/L yeast extract, 20 g/L dextrose, 1.0 g/L polysorbate 80, 2.0 g/L ammonium citrate, 5.0 g/L sodium acetate, 0.1 g/L magnesium sulfate, 0.05 g/L manganese sulfate, 2.0 g/L dipotassium phosphate, and 15 g/L Bacto agar [BD, Franklin Lakes, NJ]) and cultured for 24 h at 37 °C. A single colony was then inoculated in 5 mL of fresh MRS broth overnight at 37 °C. An aliquot (500 µL) of the overnight culture was resuspended in 10 mL of MRS broth and subsequently cultured at 37 °C without shaking until the culture reached an optical density (OD) of 0.7 at 600 nm as measured by a BioPhotometer (Eppendorf, Hamburg, Germany). The cells (1 mL) were centrifuged at 11,000 × g for 1 min and then resuspended in a buffer (1 mL) consisting of 0.1 M Tris-HCl (pH 7.6) and 0.3 M sucrose containing 5 mg/mL egg white lysozyme (Wako, Osaka, Japan). The mixture was incubated at 37 °C without shaking for 3 h. Then, the protoplasts were centrifuged at 7000 r.p.m. for 5 min and resuspended in DMB (5 g/L peptone, 1 g/L yeast extract, 0.1 g/L ferric citrate, 19.45 g/L NaCl, 5.9 g/L MgCl_2_, 3.24 g/L MgSO_4_, 1.8 g/L CaCl_2_, 0.55 g/L KCl, 0.16 g/L NaHCO_3_, 0.08 g/L KBr, 34 mg/L SrCl_2_, 22 mg/L H_3_BO_3_, 8 mg/L Na_2_HPO_4_, 4 mg/L Na_2_SiO_3_, 2.4 mg/L NaF, and 1.6 mg/L NH_4_NO_3_ [BD]) containing 300 µg/mL penicillin G. The penicillin was added to inhibit the regeneration of cell walls in the protoplasts. The resulting suspension (5 µL) was diluted with 1 mL of DMB containing 300 µg/mL penicillin G (Wako) and incubated at 24 °C.

### 2.2. qPCR

**Figure 1.**
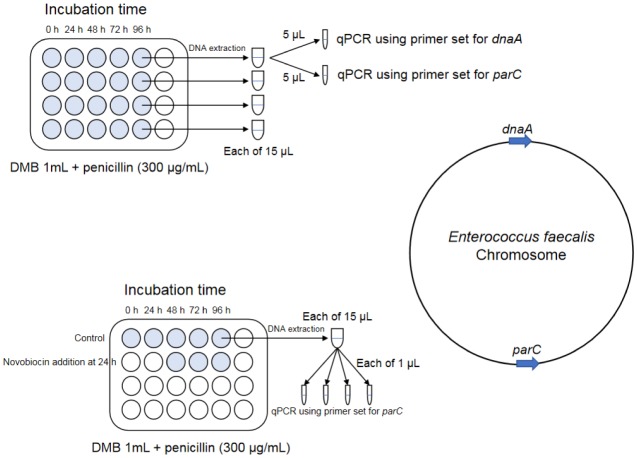
Schematic of the qPCR method and locations of *dnaA* and *parC* on the *E. faecalis* chromosome DNA. The locations of *dnaA* and *parC* were conserved among the *E. faecalis* complete chromosomes [21]–[29].

Next, DNA was extracted from the culture (1 mL) and purified using a NucleoSpin® Tissue XS kit (Macherey-Nagel GmbH & Co. KG, Düren, Germany). From the resultant 15 µL of each DNA solution, 1 or 5 µL was used for qPCR. We designed the DNA primers for real-time qPCR based on the genomic DNA sequence of *E. faecalis* in the DNA database. A comparative genomic analysis of Enterococci showed that the single-copy gene *dnaA* (encoding chromosomal replication initiator protein DnaA) locates near the replication initiation site [20]. On the *E. faecalis* genome, the single-copy gene *parC* (encoding DNA topoisomerase IV subunit A) locates at the most distant region from *dnaA*. These *dnaA* and *parC* positions were conserved among the complete chromosomes of *E. faecalis* [21]–[29]. Thus, we estimated that *parC* locates near the replication termination site. We designed *dnaA* (5′-TGAATCAACCAGATGCCAAA-3′ and 5′-CCGAAATTGTTCGGATGTCT-3′) and *parC* (5′-GTACAACGCCGCATTCTCTT-3′ and 5′-CCCCATAATGTTTCCGACAG-3′) PCR primers. The DNA was amplified using a FastStart Essential DNA Green Master kit (Roche) on a LightCycler® Nano system (Roche). qPCR was performed at 95 °C for 600 s followed by 45 cycles of denaturation (95 °C for 10 s), annealing (55 °C for 10 s), and extension (72 °C for 15 s). After extension, a melting curve analysis was performed from 60 °C–95 °C at 0.1 °C/s to confirm that nonspecific products had not been generated. The quantification cycle (Cq) values were obtained using LightCycler® Nano Software (Roche) (Figure 1).

### 2.3. Cell size measurement

Phase-contrast microscopy images of the protoplasts were obtained using an Olympus CKX41 (Tokyo, Japan). The cell sizes were measured using cellSens Standard 1.11 imaging software (Olympus).

### 2.4. Novobiocin treatment

Novobiocin, an inhibitor of DNA replication, was added to the incubation medium at 24 h at a final concentration of 50 µg/mL. Then, we measured the DNA amount in the cells and observed the cell enlargement at 24 (addition of novobiocin), 48, 72, and 96 h of incubation.

## 3. Results and discussion

### 3.1. Growth of native forms of E. faecalis

**Figure 2.**
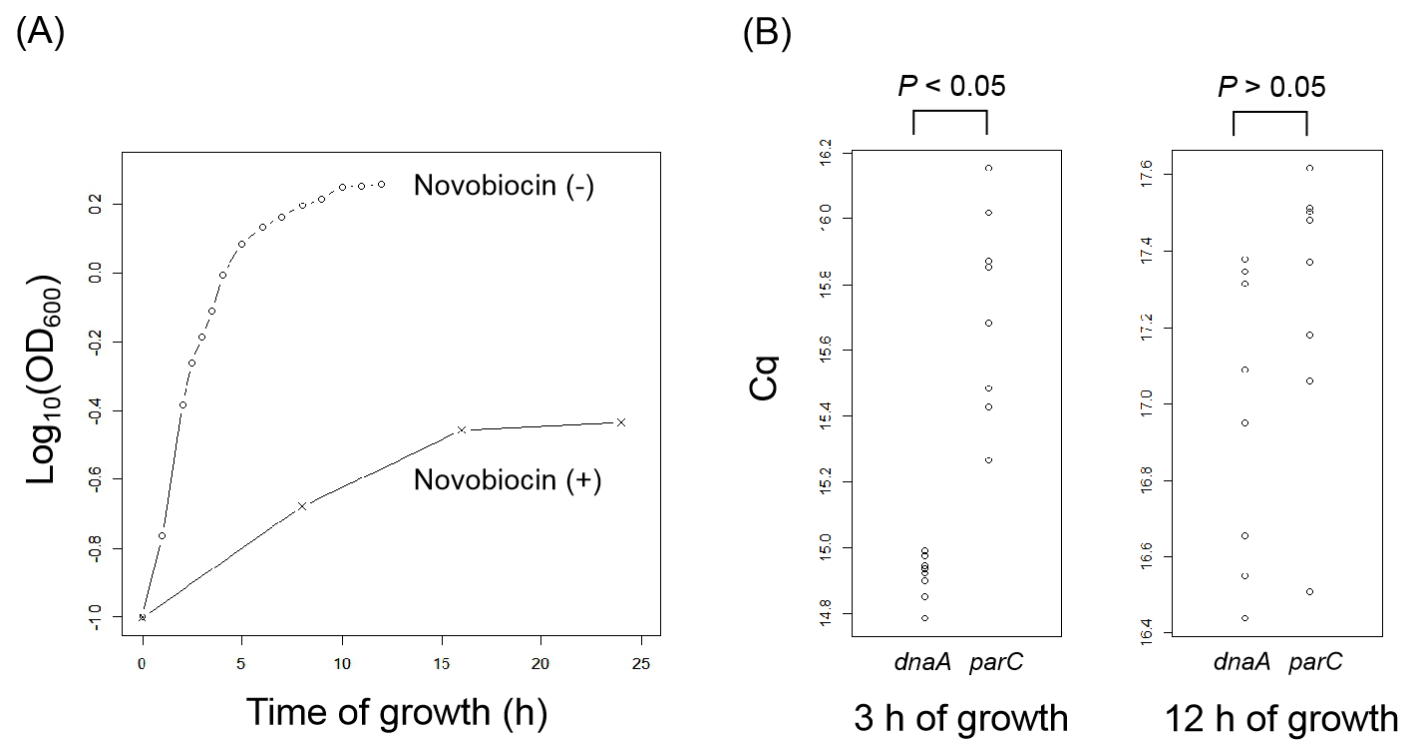
(A) Growth of normally divided cells of *E. faecalis* incubated at 37 °C in MRS broth in the absence and presence of novobiocin. The OD was measured at 600 nm. (B) Cq values at 3 h (log phase) and 12 h (stationary phase) of *E. faecalis* growth. Two qPCR primer sets (for *dnaA* and *parC*) were used. The *dnaA* and *parC* locate near the *E. faecalis* chromosome replication initiation and termination sites, respectively.

Normally divided cells of *E. faecalis* underwent a log phase before 6 h of incubation was reached (Figure 2A). In addition, novobiocin inhibited growth of normally divided cells of *E. faecalis* (Figure 2A). A significant difference in Cq values between qPCRs for *dnaA* and *parC* was observed (*p* < 0.05) in the protoplasts at 3 h (log phase) of growth. However, it was not observed (*p* > 0.05) at 12 h (stationary phase) (Figure 2B). Considering that *dnaA* locates near the replication initiation site on the *E. faecalis* chromosomal DNA and *parC* locates near the replication termination site, DNA replication activities in the log phase of growth were more than in the stationary phase. Those results were consistent with the previous study of other bacteria [30].

### 3.2. Cell enlargement of E. faecalis protoplasts

**Figure 3.**
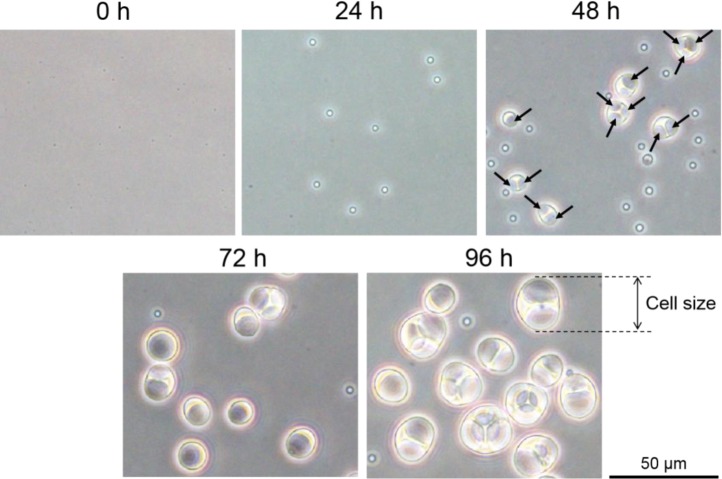
Phase-contrast microscopy images of *E. faecalis* protoplast enlargement in DMB containing penicillin. Arrows at 48 h indicate the vacuole-like structures.

**Figure 4.**
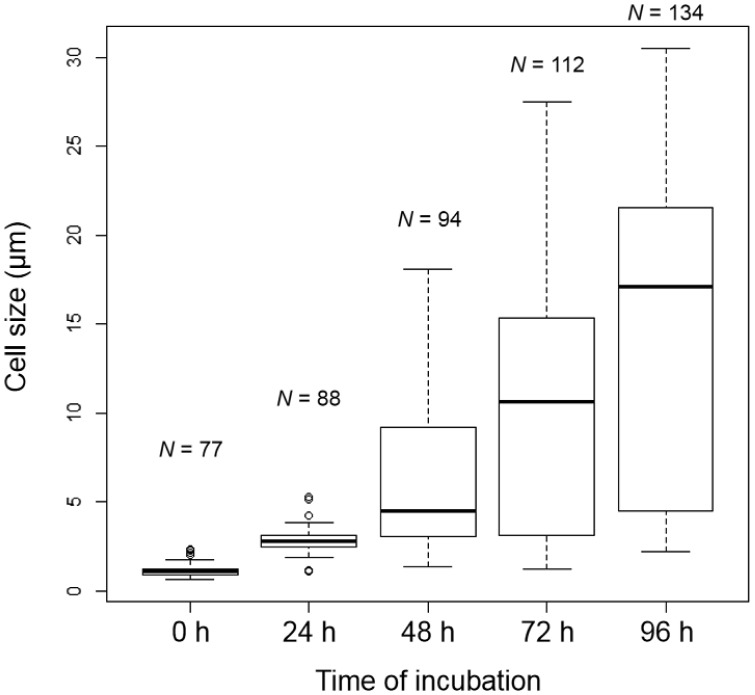
Boxplots of *E. faecalis* protoplast cell size in DMB containing penicillin. The protoplast cell size was determined using phase-contrast microscopy.

The cell size of *E. faecalis* protoplasts increased in DMB containing penicillin (Figures 3, 4). Vacuole-like structures were generated between 24 and 48 h of incubation (Figure 3). Vacuoles or vacuole-like structures have been frequently observed in enlarged bacterial spheroplasts or protoplasts, and their shape is maintained [11]–[17]. In our experiment, the enlarged vacuole-like structures in the *E. faecalis* protoplasts proceeded to press against the plasma membrane, and the cell could not maintain its spherical shape (Figure 3). This characteristic of the vacuole-like structures in enlarged *E. faecalis* protoplasts is unique.

**Table 1.** *P* values in pairwise *t* test among Cq values in Figure 2.

	*dnaA*	*parC*	*dnaA*	*parC*	*dnaA*	*parC*	*dnaA*	*parC*	*dnaA*
	0 h	0 h	24 h	24 h	48 h	48 h	72 h	72 h	96 h
*parC* 0 h	0.214								
*dnaA* 24 h	1	0.050							
*parC* 24 h	1	1	0.877						
*dnaA* 48 h	0.032	0.003	0.013	0.050					
*parC* 48 h	0.233	0.007	0.233	0.186	0.033				
*dnaA* 72 h	0.006	0.001	0.013	0.010	0.877	0.032			
*parC* 72 h	0.082	0.013	0.183	0.050	1	0.877	0.426		
*dnaA* 96 h	0.009	0.001	0.003	0.018	0.003	0.003	0.582	0.131	
*parC* 96 h	0.013	0.001	0.004	0.027	1	0.016	1	0.877	0.038

### 3.3. DNA amount increased during enlargement of E. faecalis protoplasts

**Figure 5.**
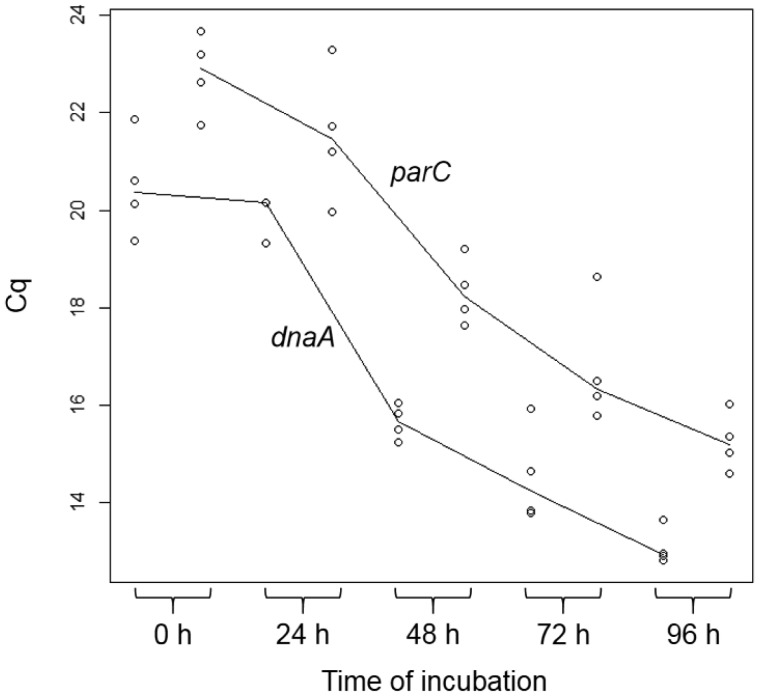
Cq values at 0, 24, 48, 72, and 96 h of *E. faecalis* protoplast incubation. Two qPCR primer sets (for *dnaA* and *parC*) were used. The *dnaA* and *parC* locate near the *E. faecalis* chromosome replication initiation and termination sites, respectively.

The Cq values significantly decreased during the course of the incubation (Figure 5, Table 1). Thus, the protoplasts of *E. faecalis* underwent DNA replication during the enlargement incubation. The Cq values of the qPCRs for *dnaA* were lower than those for *parC* at each incubation time (Figure 5). Notably, those for *dnaA* were significantly lower (*p* < 0.05) than those for *parC* at 48 and 96 h of incubation (Table 1). This might show that DNA replication was maintained at a high level during the enlargement incubation. The difference between the Cq values at 0 and 96 h of the cell enlargement incubation was 8–9 (Figure 5), indicating that the DNA amount in the *E. faecalis* protoplasts at 96 h of incubation was 200–500 times higher than that at 0 h.

Interestingly, although cell size seemed to constantly increase (Figures 3, 4), the DNA replication speed (rate) was not constant (Figure 5). The replication speed was the highest between 24 and 48 h of incubation. Thus, there was a decrease of approximately 2 Cq values between 24 and 48 h (Figure 5). On the other hand, there was a decrease of approximately 1 Cq value between the other 24 h intervals (Figure 5). Based on the cell observations, vacuole-like structures were frequently observed after 24 h of incubation (Figure 3). This suggests that DNA replication of *E. faecalis* protoplasts may be related to vacuole-like structure generation. If chromosome DNA is associated with the plasma membrane [3]–[6], additional DNA may cause membrane rearrangements that lead to vacuole formation and expansion.

### 3.4. Effect of novobiocin on protoplast enlargement, DNA replication, and vacuole-like structure generation

**Figure 6.**
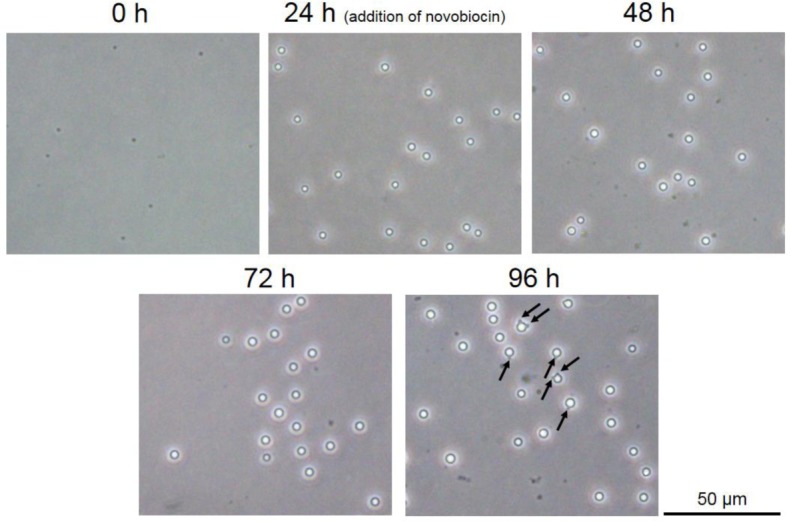
Phase-contrast microscopy images of *E. faecalis* protoplast enlargement in DMB containing penicillin (novobiocin addition at 24 h of incubation). Cell enlargement was strongly inhibited following novobiocin addition. Arrows at 96 h indicate protrusions of the vacuole-like structures.

**Figure 7.**
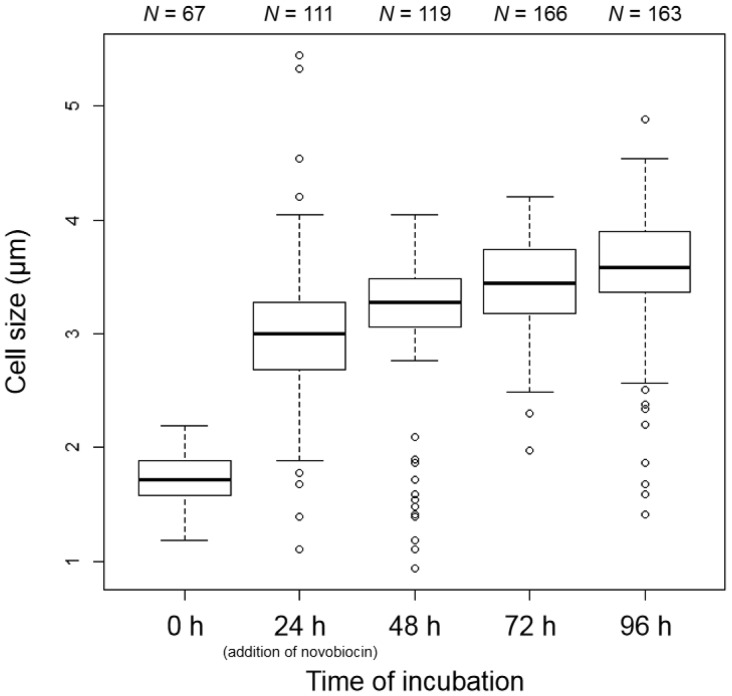
Boxplots of *E. faecalis* protoplast cell size in DMB containing penicillin (novobiocin addition at 24 h of incubation). The protoplast cell size was determined using phase-contrast microscopy. As a result of a pairwise *t*-test among cell sizes at each time point of *E. faecalis* protoplast incubation with Holm adjustment using R, a significant difference among all samples was obtained.

**Figure 8.**
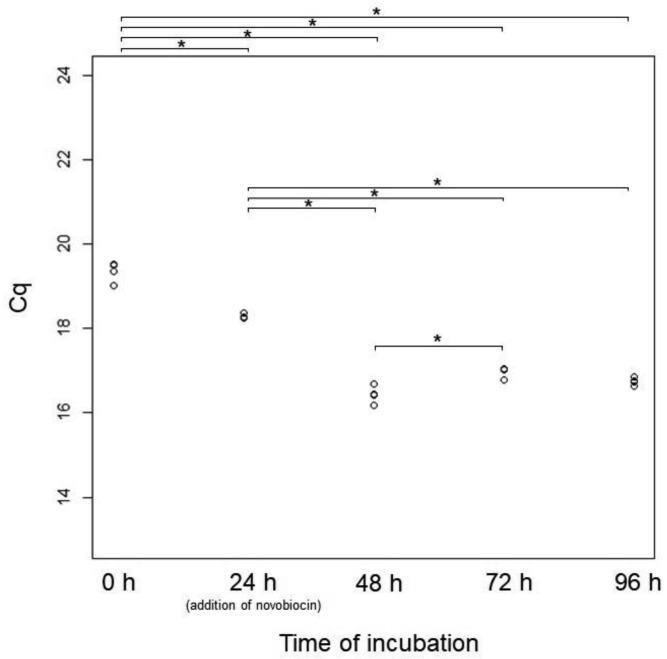
Cq values at 0, 24, 48, 72, and 96 h of *E. faecalis* protoplast incubation (novobiocin addition at 24 h of incubation). The qPCR primer set for *parC* was used. The final concentration of novobiocin was 50 µg/mL. We performed a pairwise *t*-test among Cq values at each time point of *E. faecalis* protoplast incubation with Holm adjustment using R software. Asterisks indicate *p* < 0.05.

We added the DNA replication inhibitor novobiocin to the medium at 24 h of incubation. Novobiocin inhibits DNA gyrase activity [31],[32]. Following novobiocin addition, cell enlargement was strongly inhibited (Figures 6 and 7). However, the cells tended to grow very slowly (Figure 7). At 96 h of incubation, some protrusions were observed (Figure 6). These results suggest that the *E. faecalis* protoplasts were alive after novobiocin addition. Additionally, vacuole-like structures were not observed (Figure 6). Although normally divided cells did not form colonies on the agar medium in the presence of novobiocin, they formed colonies in the absence of novobiocin after incubation in the presence of novobiocin, indicating that the cells were alive in the presence of novobiocin (Figure S1). In addition, the *E. faecalis* protoplasts enlarged after novobiocin removal (Figure S2). Although the chromosomal DNA was synthesized between 24 and 48 h of incubation, the Cq values were almost unchanged after 48 h (Figure 8). This indicates that DNA replication stopped after 48 h of incubation. It is suggested that novobiocin affected DNA gyrase soon after its addition, but it may not have been effective in blocking the first round of DNA replication after the addition of novobiocin [33]. Thus, DNA replication (DNA biosynthesis) may be associated with biosynthesis of the plasma membrane and vacuole-like structures' membranes in *E. faecalis* protoplasts. This strongly suggests that DNA replication is required for the cell enlargement of *E. faecalis*.

In native forms, DNA replication is associated with cell surface biosynthesis for normal cell division. If bacterial protoplasts maintain such a system (the relationship between DNA replication and cell surface biosynthesis), chromosomal DNA replication (biosynthesis) might be associated with plasma membrane biosynthesis in *E. faecalis* protoplasts. Additionally, our findings strongly suggest that plasma membrane biosynthesis may be associated with the biosynthesis of the vacuole-like structures' membranes. To elucidate the relationship between vacuole formation and protoplast enlargement, further research is required.

In conclusion, we demonstrated that DNA replication was occurring while the *E. faecalis* protoplasts were enlarging. Novobiocin inhibited cell enlargement as well as DNA replication. Thus, chromosomal DNA replication plays an important role in the enlargement of *E. faecalis* protoplasts.
