# Supplementary material for: DNA replication and cell enlargement of Enterococcus faecalis protoplasts
Source: AIMS Microbiol. 2019 Oct 25;5(4):347–57. doi: 10.3934/microbiol.2019.4.347 (PMC6946640; doi:10.3934/microbiol.2019.4.347)
Supplement: Supplementary file 1 [file microbiol-05-04-347-s001.pdf]

AIMS Microbiology
http://www.aimspress.com/journal/microbiology

AIMS Microbiology, 5(4): 347–357.
DOI: 10.3934/microbiol.2019.4.347
Received: 19 August 2019
Accepted: 23 October 2019
Published:

Research article

DNA replication and cell enlargement of *Enterococcus faecalis* protoplasts

Satoshi Kami†, Rintaro Tsuchikado†, Hiromi Nishida*

Department of Biotechnology, Toyama Prefectural University, 5180 Kurokawa, Imizu, Toyama 939-0398, Japan.

† These two authors contributed equally.

* **Correspondence:** Email: hnishida@pu-toyama.ac.jp.

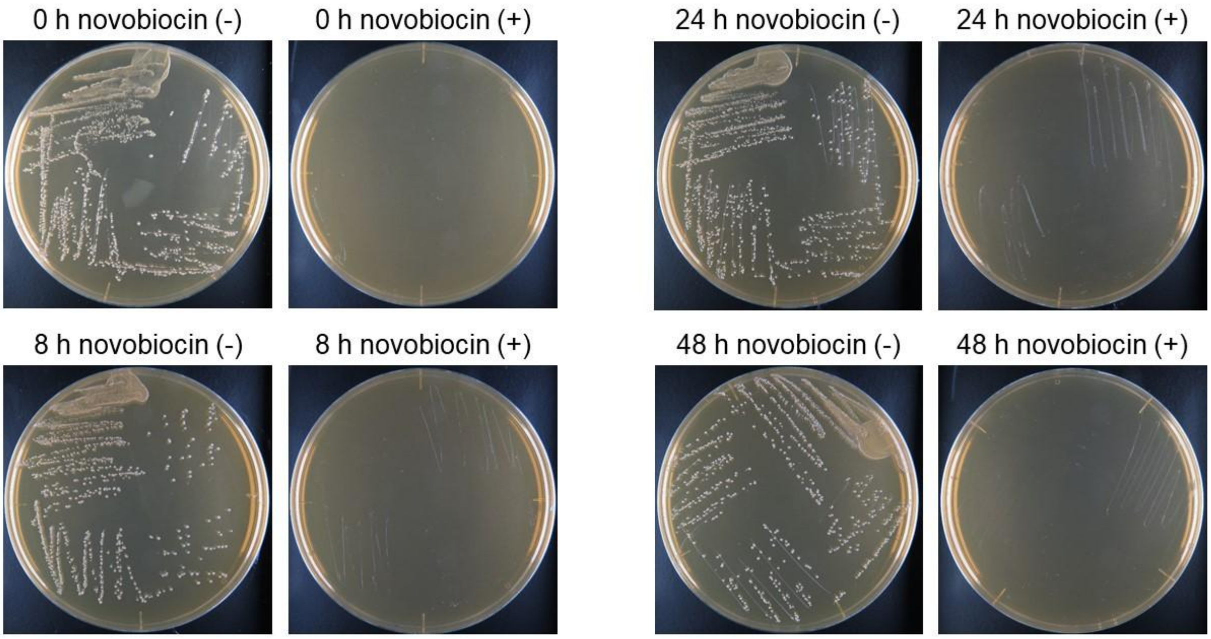

Figure S1. Normally divided cells of *E. faecalis* were incubated in MRS liquid medium containing 50 μg/ml novobiocin. The cells at 0 h, 8 h, 24 h, and 48 h of incubation were inoculated on the agar medium with/without novobiocin.

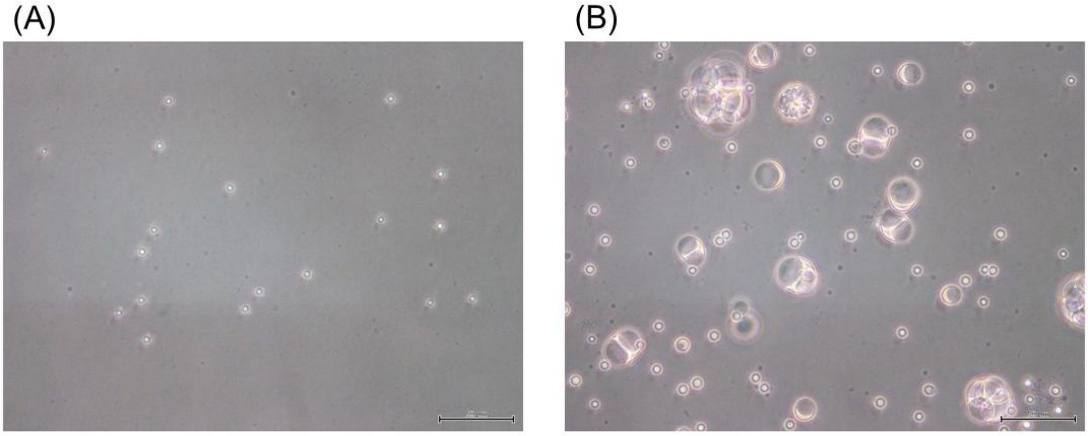

Figure S2. Phase-contrast microscopy images of *E. faecalis* protoplasts. (A) Cells at 144 h of incubation (novobiocin addition at 24 h). (B) Cells at 144 h of incubation (novobiocin addition at 24 h and removal at 48 h). Novobiocin was added to the incubation medium at 24 h at a final concentration of 50 µg/mL. Concentration of novobiocin was diluted 50 times (1 µg/mL) at 48 h.

AIMS Press

© 2019 the Author(s), licensee AIMS Press. This is an open access article distributed under the terms of the Creative Commons Attribution License (http://creativecommons.org/licenses/by/4.0)
